# Development and validation of an advanced *ex vivo* brain slice invasion assay to model glioblastoma cell invasion into the complex brain microenvironment

**DOI:** 10.3389/fonc.2023.976945

**Published:** 2023-01-26

**Authors:** Lisa R. Decotret, Rocky Shi, Kiersten N. Thomas, Manchi Hsu, Catherine J. Pallen, Kevin L. Bennewith

**Affiliations:** ^1^ Department of Integrative Oncology, BC Cancer, Vancouver, BC, Canada; ^2^ Department of Pathology and Laboratory Medicine, Faculty of Medicine, University of British Columbia, Vancouver, BC, Canada; ^3^ Interdisciplinary Oncology Program, University of British Columbia, Vancouver, BC, Canada; ^4^ Michael Cuccione Childhood Cancer Research Program, BC Children’s Hospital Research Institute, Vancouver, BC, Canada; ^5^ Department of Pediatrics, Faculty of Medicine, University of British Columbia, Vancouver, BC, Canada

**Keywords:** organotypic brain slice culture, brain microenvironment, invasion, metastasis, glioblastoma multiforme, confocal microscopy

## Abstract

Organotypic cultures of murine brain slices are well-established tools in neuroscience research, including electrophysiology studies, modeling neurodegeneration, and cancer research. Here, we present an optimized *ex vivo* brain slice invasion assay that models glioblastoma multiforme (GBM) cell invasion into organotypic brain slices. Using this model, human GBM spheroids can be implanted with precision onto murine brain slices and cultured *ex vivo* to allow tumour cell invasion into the brain tissue. Traditional top-down confocal microscopy allows for imaging of GBM cell migration along the top of the brain slice, but there is limited resolution of tumour cell invasion into the slice. Our novel imaging and quantification technique involves embedding stained brain slices into an agar block, re-sectioning the slice in the Z-direction onto slides, and then using confocal microscopy to image cellular invasion into the brain tissue. This imaging technique allows for the visualization of invasive structures beneath the spheroid that would otherwise go undetected using traditional microscopy approaches. Our ImageJ macro (BraInZ) allows for the quantification of GBM brain slice invasion in the Z-direction. Importantly, we note striking differences in the modes of motility observed when GBM cells invade into Matrigel *in vitro* versus into brain tissue *ex vivo* highlighting the importance of incorporating the brain microenvironment when studying GBM invasion. In summary, our version of the *ex vivo* brain slice invasion assay improves upon previously published models by more clearly differentiating between migration along the top of the brain slice versus invasion into the slice.

## 1 Introduction

Glioblastoma multiforme (GBM) is a late-stage astrocytoma brain tumour that is considered the most aggressive form of brain cancer. GBM tumours account for 12-15% of brain tumours diagnosed in North America (1). The incidence of GBM is 4 per 100,000 people and more commonly occurs in adult males than females (2). Only 4-7% of patients diagnosed with GBM will live more than 5-years (1, 3, 4). Despite decades of research, few advancements have been made to improve survival outcomes for GBM patients, which is partially attributed to a lack of appropriate model systems that accurately recapitulate the complex brain environment.

Brain tissue is a unique environment that contains high levels of astrocytes, proteoglycans/glycoproteins and hyaluronic acid (HA), while consisting of low levels of fibrous proteins (laminin, fibronectin, collagens). Brain tissue is softer than other organs, with the majority of fibrous proteins restricted to the basement membrane surrounding the vasculature (5, 6). Typical *in vitro* models of cancer cell invasion use a cocktail of extracellular matrix (ECM) proteins such as laminin and collagen (*i.e.* Matrigel, gelatin, etc.) that lack other brain specific components and thus do not represent a physiologically relevant substrate to model brain tumour cell invasion. Another unique feature of GBM invasion is that GBM cells seldom intravasate into the vasculature. As early as 1938, researcher Hans Joachum Scherer found glioma cells migrate along brain structures later termed “Scherer’s structures” (7). It is now well understood that brain tumour cells preferentially grow and invade along pre-existing structures including myelinated axons, blood vessels, and white matter tracts in the parenchyma, but can invade through the fluid-filled perivascular space surrounding the vasculature as well (8–11).

The organotypic brain slice invasion assay was first developed by Ohnishi and colleagues in 1998 and has been seminal for studying mechanisms of glioblastoma invasion using a biologically relevant scaffold (12). Several groups have since published a version of the *ex vivo* brain slice model using various methods for implanting tumour cells and imaging cellular motility. For instance, some groups use the technique of seeding tumour spheroids on top of brain sections (13–15), implanting tumour spheroids within the slice using a blunt-edge needle (16), or simply seeding tumour cells on top of the brain slice in a small droplet of medium (17). Other groups use alternative approaches such as cutting the brain slices in half and seeding tumour cells onto the membrane to create a “cell field” between the two hemispheres (18) or mixing tumour cells with ECM (*i.e.* Matrigel) and seeding the mixture into a cell spacer directly adjacent to the edge of the brain slice (19). Imaging strategies are also varied, ranging from taking a single image of the brain slice-spheroid interface to taking z-stack images through the brain slice on a confocal microscope. Importantly, many of these previous reports do not distinguish between migration along the top of the slice and invasion of cells into the slice.

Herein, we describe a universal technique for implanting tumour spheroids onto *ex vivo* brain slices without the use of Matrigel or other exogenous matrices that are not present in the brain environment. We also outline an improved imaging strategy for visualizing and quantifying cellular invasion into the brain slice. Our protocol involves embedding brain slices containing tumour spheroids into agar blocks that are re-sectioned onto slides to allow for the visualization of invasive projections below the tumour spheroids that would otherwise go undetected. The refinement of physiologically relevant models of GBM invasion will help to advance the development and testing of therapeutic strategies to reduce GBM cell motility and improve patient outcomes.

## 2 Materials and equipment

### 2.1 Reagents

- C57BL/6J mice, 6-weeks-old, male.
- Cell lines: Human glioblastoma cell lines LN229 (Cat.No. CRL-2611) and LN18 (Cat.No. CRL-2610) were purchased from ATCC (www.atcc.org). LN229 and LN18 cells were transfected with pLenti-CMV-GFP-Blast (659-1) lentiviral vector plasmid (Addgene, Cat.No. 17445) and GFP-expressing cells were selected with 8 µg/mL blasticidin-containing medium for 3 days (**Supplementary Methods**).
- LN229 and LN18 GFP-expressing cells were grown in 8 µg/mL blasticidin-containing medium for 3 days to select for blasticidin-resistant cells, then maintained in cell culture medium.
- Cell culture medium: Dulbecco’s modified Eagle’s medium (DMEM) (Cytiva Hyclone, Cat.No. SH30243) containing 5% fetal bovine serum (FBS).
- Brain slicing solution: 1X Hanks’ Balanced Salt Solution (HBSS) (ThermoScientific, Cat.No, 14025134), 1% penicillin-streptomycin (ThermoScientific, Cat.No. 15140122), 4 mM magnesium chloride, and 5 mM D-glucose (dextrose). Aerate the solution with microbubbles of carbogen gas (95% O_2_, 5% CO_2_) for a minimum of 30 minutes.
- Brain slice culture medium: Dulbecco’s modified Eagle’s medium (DMEM)/F-12 (ThermoScientific, Cat.No. 1133032) containing 25% fetal bovine serum (FBS) and 1% penicillin-streptomycin (ThermoScientific, Cat.No, 15140122).
- 1X PBS (ThermoFisher, Cat.No. 21300058)
- Agar: 1% (generating spheroids) and 4% (embedding brain slices) agar dissolved in dH_2_0. Heat agar solution in the microwave for 1-2 minutes until dissolved, then autoclave to sterilize.
- 10% alamarBlue™: Dilute alamarBlue™ cell viability reagent (Invitrogen, DAL1100) in brain slice culture medium.
- 4% Paraformaldehyde (PFA): Dilute 16% PFA in 1X PBS (Electron Microscopy Sciences, Cat.No. 50-980-487).
- Permeabilization buffer: 0.2% Triton-X-100 in 1X PBS.
- Blocking buffer: 3% FBS, 3% bovine serum albumin (BSA) (Sigma, Cat.No. A7906-100G), 0.2% Triton-X-100 in 1X PBS.
- Primary and secondary antibodies: anti-GFAP (Abcam, Cat.No. ab7260), anti-α-SMA (Abcam, Cat.No, ab7817), Alexa Fluor 594-conjugated goat anti-mouse IgG (Molecular Probes, Cat. No. A11032), and Alexa Fluor 647-conjugated goat anti-rabbit IgG (Life Technologies, Cat.No. A21245).

### 2.2 Equipment

- Dissection tools: scissors (curved), dissecting forceps (2)
- Metal spatula, flat (VWR, Cat.No. 82027-532)
- Leica VT1000 S vibrating blade microtome, Vibratome (Leica Biosystems, Cat.No. VT1000S)
- Carbogen gas tank (95% O_2_, 5% CO_2_) and pressure regulator
- Micro-bubble diffuser (Product number: Naludo-NL138)
- Double-edge stainless steel microtome blades (Ted Pella, Cat.No. 121-6)
- Razor blades, 0.30 mm (VWR, Cat.No. 55411-055)
- Loctite quick set instant 404 adhesive (Henkel, Cat.No. 135465)
- Millicell cell culture inserts (Millipore, Cat.No. PICM03050)
- Round-bottom 96-well plates (Sarstedt, Cat.No. 83.3925)
- p200 pipette tips
- Parafilm (VWR, Cat.No. 52858-032)
- Disposable base molds, 15 x 15 x 5 mm (FisherScientific, Cat.No. 22-363-553)
- Superfrost Plus Micro Slice (VWR, Cat.No. 48311-703).
- Glass coverslips, 50 x 24 mm (FisherScientific, Cat.No. 12-548-5M)
- Kimwipes (VWR, Cat.No. CA21905-026)
- Quick-dry nail polish
- Laser scanning confocal microscope (Zeiss LSM 800 with Airyscan detector, Zen 2 Imaging Software, 20X/0.8 air objective lens, no correction collar, 35 μm pinhole size)
- “BraInZ” ImageJ macro to measure GBM tumour cell invasion into *ex vivo* brain slices in the Z direction. https://github.com/ldecotret/BraInZ.git

### 2.3 Methods

**Figure 1:**
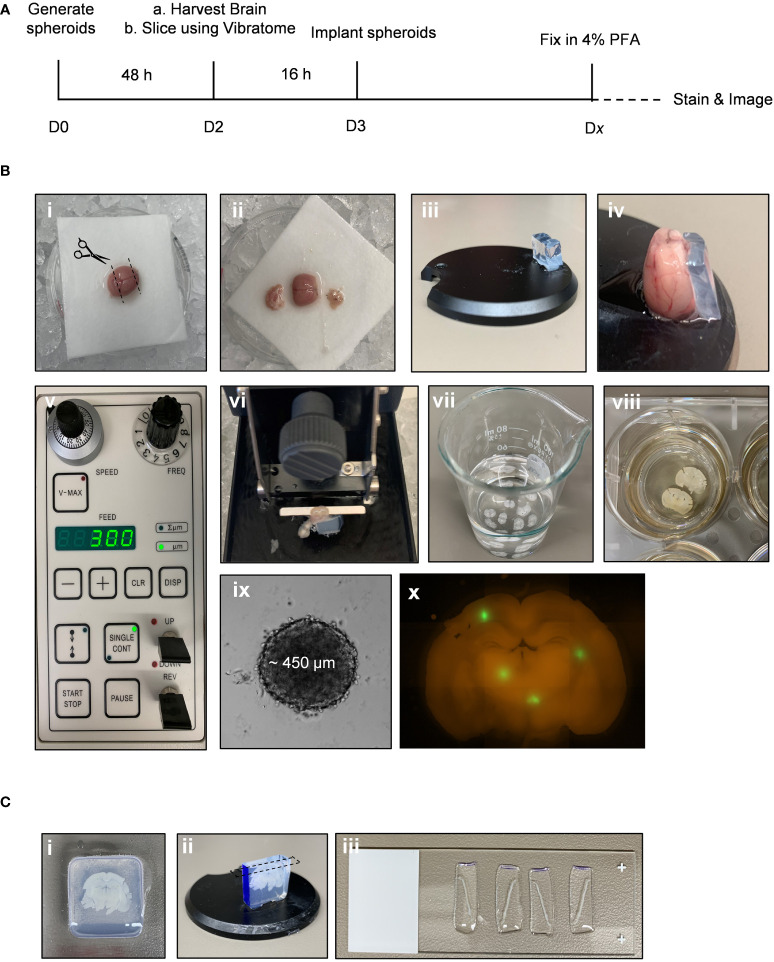
*Ex vivo* brain slice spheroid invasion assay workflow. **(A)** Summary of the *ex vivo* brain slice invasion assay experimental timeline. **(B)** Experimental steps required to preform the *ex vivo* brain slice invasion assay: (*i*) Whole brains were harvested from 6-week-old male C57BL/6J mice and (*ii*) the olfactory bulb and cerebellum were removed. (*iii*) A block of solidified 5% agar was glued to the edge of the vibratome disc directly opposite the disc indentation. (*iv*) The isolated cerebral cortex was then glued directly to the vibratome disc such that it is leaning against the agar block with the forebrain facing upwards. (*v*) A Leica vibratome (VT100S) was used to generate brain slices (speed: 0.15 mm/s; frequency: 80 Hz). (*vi*) The vibratome disc containing the brain was submerged in ice-cold carbogenated slicing solution (95% O_2_, 5% CO_2_) and (*vii*) 300 µm brain slices were collected into a beaker containing the cold slicing solution. (*viii*) Once all brain slices are collected, 2-3 brain slices were transferred onto permeable cell culture inserts submerged in 25% FBS-containing DMEM/F12 medium. (*ix*) GBM spheroids were generated and (*x*) implanted onto the brain slices *ex vivo*. **(C)** Experimental steps required to generate brain slice z-sections. (*i*) A brain slice containing GFP-positive GBM spheroids was transferred to a plastic mold and embedded in 4% agar. (*ii*) Once the agar is solidified, the agar block is removed form the cryomold, the orientation is marked with a sharpie marker, and the agar block is glued to the vibratome disc. (*iii*) The vibratome was used to generate 200 µm z-sections. Each z-section was collected and mounted onto microscope slices for imaging.

A general experimental timeline can be found in **Figure 1A**. If any issues arise, a troubleshooting guide listing common issues and possible solutions has been provided (**Table 1**).

**Table 1:** Troubleshooting guide for the *ex vivo* brain slice spheroid invasion assay.

Issue	Possible Causes	Solutions
Large cuts along sides of the brain slices.	Cutting into the side of the brain during removal.	Use curved scissors to cut along the side of the skull pointing outward to avoid the brain.
Brain slices fall apart during transfer steps.	Incorrect placement of the spatula.	Use a spatula with a wide, flat end to scoop up the brain slice. Be sure to place the spatula directly in the center of the slice before lifting.
Fragmented z-sections.	(i) The agar block was glued to the platform at an angle resulting in the blade skipping. (ii) One side of the brain slice is too close to the edge of the agar block. (iii) The z-section is too thin and is falling apart.	(i) Remove the agar block from the platform and re-glue such that the top of the block is perfectly perpendicular to the platform. (ii) Ensure there is sufficient agar on the top and bottom of the brain slice when embedding into agar block. (iii) Increase the thickness of each z-slice.
Spheroid appears too small.	Imaging the edge of the spheroid.	When z-sectioning the brain slice, parts of the same spheroid will appear in multiple z-sections. Thus, if the spheroid appears too small, image the surrounding z-sections to get a more representative sample.
Spheroid is too large and fills the entire z-section of brain slice.	(i) Spheroid too large for the thickness of the brain slice. (ii) Tumour type is highly invasive. (iii) Slice is too thin for the size of the spheroid.	(i) Decrease the initial size of the spheroids. (ii) Decrease the length of the invasion assay. (iii) Increase the slice thickness to 400 µm to create more space.
Spheroid appears out of focus when imaging.	A bubble formed around the spheroid when placing coverslip over z-sections.	Remove the coverslip, blot excess PBS with a Kimwipe™, and replace coverslip to remove bubbles.

#### 2.3.1 Day 0 - Generating tumour spheroids

1. Using a multichannel pipette, add 100 µL 1% sterile agar per well of a round-bottom 96-well plate to coat the bottom of the plate. Quickly aspirate the agar solution and allow plate to air dry for 30 minutes.
2. Seed 1.0 x 10^4^ cells per well in 100 µL complete cell culture medium, centrifuge plate at 130 RCF for 3 minutes, and then incubate at 37°C and 5% CO_2_ for 3 days.

#### 2.3.2 Day 2 - Harvesting brain slices

1. Prepare fresh brain slice culture medium and slicing solution prior to the start of each experiment.
2. Transfer 1 Millicell cell culture insert per well of a 6-well dish. Condition membranes by adding 1 mL brain slice culture medium to the bottom well and 1 mL medium to the top of the insert, incubate at 37°C and 5% CO_2_ for 1 hour.
3. Harvest naïve brains from 6-week-old male C57BL/6J mice. Once the brain has been removed from the skull, immediately submerge in slicing solution for at least 1 minute.
*NOTE: For improved brain viability, remove 1 brain at a time and keep the total dissection time to under 5 minutes.*
4. Isolate the cerebral cortex by removing the cerebellum and olfactory bulb using a razor blade (**Figure 1B** *i, ii*).
5. Adhere a block of 5% agar, approximately 2 cm (L), ½ cm (W), 1 cm (H) in size, directly to the specimen disc using quick dry superglue. Then glue the cerebral cortex directly to the specimen disc using quick dry superglue, forebrain facing upwards, directly against the agar block (**Figure 1B** *iii, iv*).
6. Set up the vibratome: Set the speed to 3 (0.15 mm/s), the frequency to 8 (80 Hz), and the feed to 300 µm (**Figure 1B** *v*). Set the clearance angle of the blade holder to 15 degrees.
7. Transfer the specimen disc to the vibratome reservoir containing slicing solution that has been aerated with carbogen (95% O_2_, 5% CO_2_). Fill outer reservoir with ice (**Figure 1B** *vi*).
8 Generate 300 µm brain slices. Transfer each slice using a flat, metal spatula to a 50 mL beaker containing 25 mL of slicing solution (**Figure 1B** *vii*).
9 Working in a sterile biological safety cabinet, transfer brain slices to the previously prepared 6-well dish containing brain slice medium. A maximum of 3 slices per membrane is recommended to avoid overlapping the slices. Remove excess medium from the top of the insert such that brain slices no longer float or move around in the medium. Incubate brain slices at 37°C and 5% CO_2_ overnight (**Figure 1B** *viii*).

#### 2.3.3 Day 3 – Implanting tumour spheroids onto brain slices

1. On day 3, the tumour spheroids should be fully formed and have an approximate average diameter of 450 µm ± 30 SD (**Figure 1B** *ix*).
2. To implant the GFP-expressing spheroids onto a brain slice, use a p200 pipette + tip to pick up a single spheroid in a small amount of medium (~25 µL). Carefully expel the medium onto the brain slice in the exact location you wish to implant the spheroid (**Figure 1B** *x*).
3. Incubate the brain slice at 37oC and 5% CO2 for 2-5 days.

#### 2.3.4 Day 5-8* - Immunofluorescent staining

* The experimental endpoint may vary depending on the initial spheroid size and the invasiveness of different cancer cell lines.

1. At the experimental endpoint, remove medium and add 1 mL 4% PFA to the top and 1 mL 4% PFA to the bottom of each membrane. Incubate at room temperature for 2 hours and then remove PFA. Wash with 1X PBS three times for 5 minutes each.
2. Using the metal spatula, transfer brain slices to a 24-well plate with 1 brain slice per well.
3. Permeabilize brain slices with 0.5% Triton-X-100 in PBS for 30 minutes at room temperature while shaking gently.
4. Block brain slices with 3% FBS, 3% BSA, and 0.2% Triton-X-100 in PBS for 2 hours at room temperature while shaking gently. Wash slices once with 1X PBS for 5 minutes.
5. Add the primary antibodies (alone or in a cocktail) diluted in blocking buffer. Wrap plate with parafilm and incubate at 4°C while shaking gently for 2 days. Wash slices three times with 1X PBS for 5 minutes each.
6. Add secondary antibody(ies) diluted in 1X PBS and incubate overnight at 4°C while shaking. Wash slices three times with 1X PBS for 5 minutes each.

#### 2.3.5 Staining + 3 days - Re-sectioning brain slices

1. Once immunofluorescent staining is complete, immediately embed slices into agar by adding 1-2 mL hot 4% agar into the plastic mold then transferring 1 brain slice per mold using the metal spatula (**Figure 1C** *i*).
*NOTE 1: The agar solution will cool down quickly when left at room temperature. If the solution starts to solidify, microwave for an additional 20 seconds.*
*NOTE 2: The use of 4% agar is important for embedding the brain slices because the increased stiffness of the agar is required when re-sectioning the slice using the vibratome.*
2. Once the agar solution has solidified, remove block from plastic mold. If different spheroid conditions are implanted onto the same brain slice, indicate the brain slice orientation by marking one side of the agar block with a sharpie marker (**Figure 1C** *ii, blue*).
3. Glue the agar block containing a stained brain slice to the specimen disc with quick dry superglue (**Figure 1C** *ii*).
4. Fill the vibratome reservoir with ice-cold 1X PBS and submerge the specimen disc.
5. Use the vibratome to generate 200 µm sections. Transfer each section to a microscope slide, with a maximum of 6 sections per slide. Blot excess PBS using a Kimwipe, gently add a coverslip on top of the sections, and seal the edges of the coverslip with nail polish.
*NOTE: PBS was used to mount the coverslips to generate brain slice images; mounting medium was not required to produce high quality images using our method.*
6. Leave microscope slides overnight at room temperature. Protect from light.

#### 2.3.6 Staining + 4 days – Image acquisition and analysis of invasion into the brain slice

##### 2.3.6.1 Image acquisition using a confocal microscope

1. The following day, image the 200 µm sections through the coverslip using a confocal microscope. Sections must be imaged within 36 hours from slicing.
2. Images should be acquired using a 20X objective and stitching multiple images together using the “tile” function.

##### 2.3.6.2 Image analysis

*NOTE: The following steps are performed using (Fiji Is Just) ImageJ software (version 2.0.0-rc-69).

**Figure 2:**
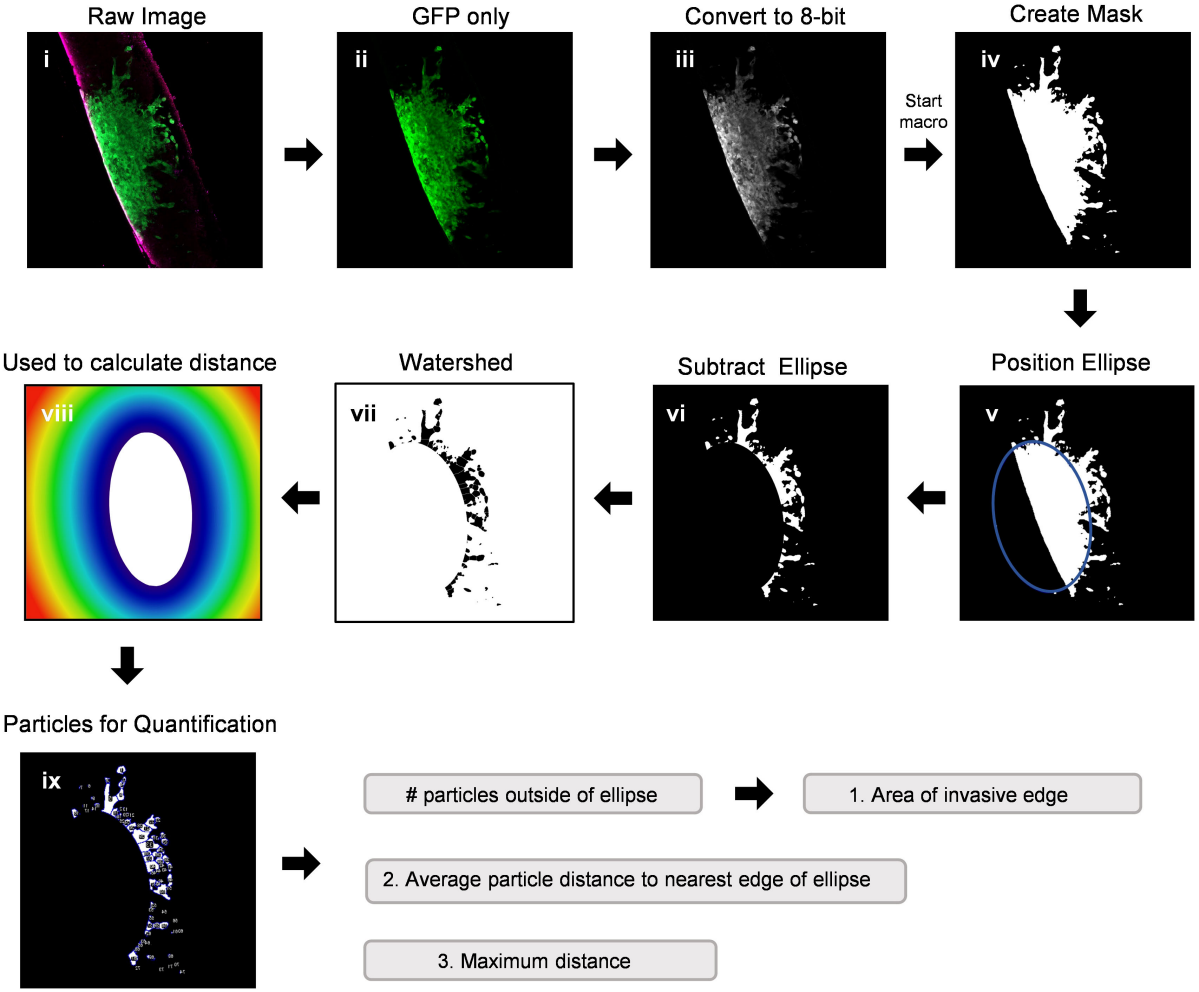
Workflow of a semi-automated ImageJ macro used to quantify invasion into *ex vivo* brain slice cultures. This ImageJ macro is comprised of 9 semi-automated steps for analyzing the number of particles, area of each particle, and distance of each particle from the nearest edge of the spheroid. (i) A raw image is split into individual channels and (ii) the GFP only channel is converted to an 8-bit image (iii). The ImageJ macro then starts by creating a mask of the binary image (iv) and the software places an ellipse over the core of the spheroid, which can be adjusted if necessary to capture the edge of the spheroid (v). The ellipse is subtracted from the image (vi) and particles outside of the spheroid core are watershed (vii). Finally, a colour map (viii) is used by the software to calculate the distance of each particle from the nearest edge of the ellipse (ix) as well as the area of each particle.

1. Open raw image file in ImageJ (**Figure 2** *i*).
2. Split channels to isolate the GFP image: Image > Color > Split Channels (**Figure 2** *ii*).
3. Convert GFP image to 8-bit: Image > Type > 8-bit (**Figure 2** *iii*).
4. Open “BraInZ” ImageJ macro: Drag and drop the .ijm file into (Fiji Is Just) ImageJ software.
5. Press run and follow macro instructions (*i.e.*, position ellipse such that ellipse outlines the solid tumour area) (**Figure 2** *iv-ix*).
6. Copy and paste the data from the “Results” window into a spreadsheet. Calculate the following: (1) Area of the invasive edge = sum of the area of particles outside of ellipse, (2) Average distance to the nearest edge of the ellipse = average the distance of each particle from the nearest edge of the ellipse, and (3) Maximum distance = greatest particle distance from the edge of the ellipse.

## 3 Results

**Figure 3:**
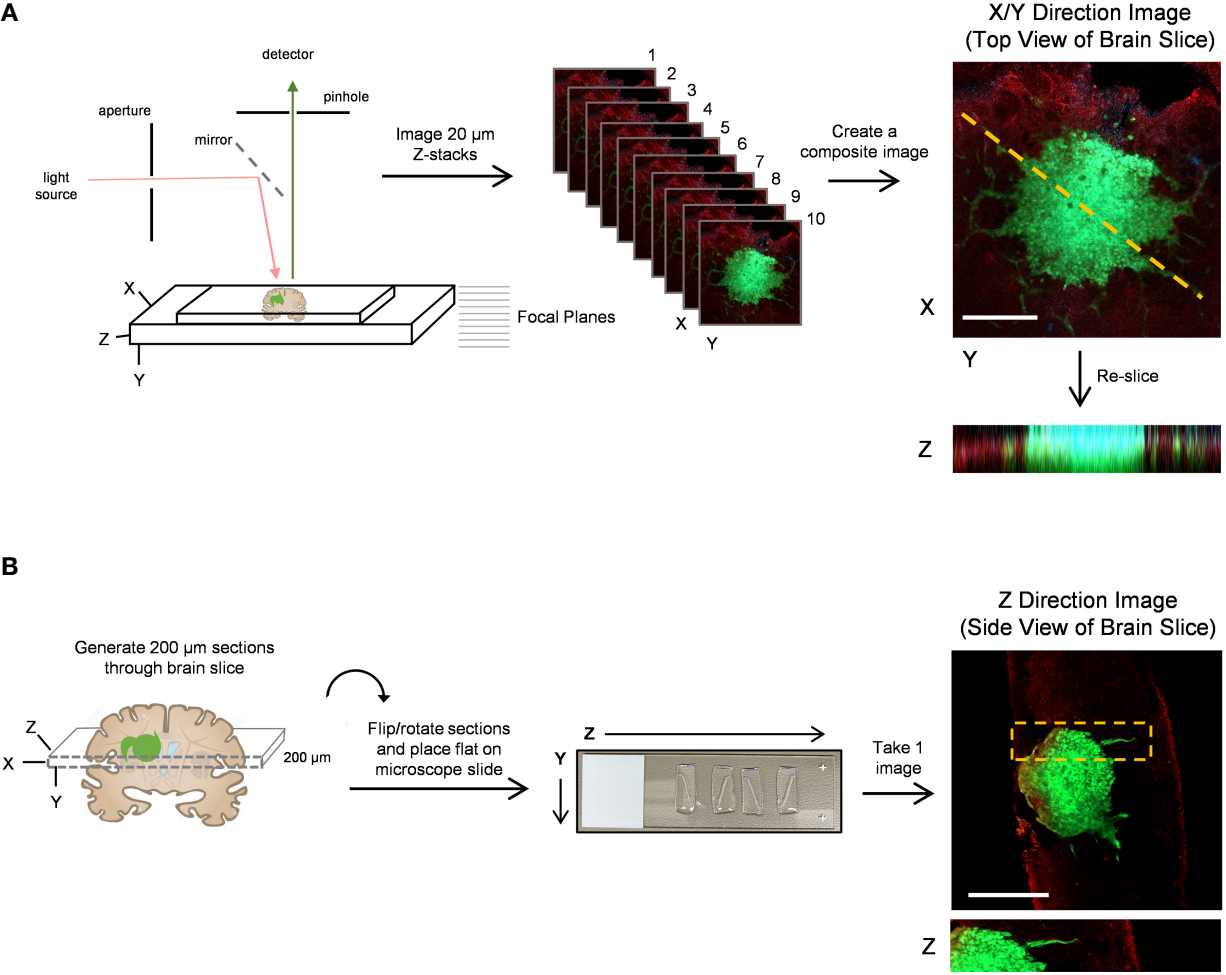
Confocal imaging techniques to improve depth of imaging. **(A)** A schematic of typical confocal microscope imaging parameters showing a whole brain slice optically sectioned at 20 µm intervals. Ten z-stack images were used to create a single composite 200 µm image of a LN18-GFP tagged spheroid invading on a brain slice in the X/Y direction. Scale bar = 500 µm. The re-slice function in ImageJ was used to extract an image in the Z-direction along the yellow dotted line. **(B)** A schematic displaying a whole 300 µm brain slice physically re-sliced on the vibratome into perpendicular 200 µm sections, which are then flipped 90°, rotated clockwise and laid flat onto a microscope slide. A single z-plane image was captured by confocal microscopy revealing a LN18-GFP tagged spheroid invading into a brain slice in the Z-direction. Scale bar = 250 µm. Brain slices were stained for GFAP to mark astrocytes (red).

Modeling GBM invasion into *ex vivo* brain slices in the Z-direction can be technically challenging, particularly when attempting to distinguish between cellular migration along the top of the slice and tumour cell invasion into the slice. Thus, we sought to improve deep tissue imaging strategies such that visualizing cellular invasion into the brain slice is possible. We first mounted a whole 300 µm brain slice containing a GFP-expressing GBM spheroid onto a microscope slide, secured it in place with a coverslip over PBS, and then used the confocal microscope Z-stack function to capture 20 µm images in 10 focal planes (**Figure 3A**). The Z-stack images were used to create a single composite image of the brain slice in the X/Y direction. The ImageJ re-slice function was then used to generate a side view of the image stack across the center of the spheroid (**Figure 3A**, yellow dotted line), which represents invasion into the brain slice in the Z-direction. Despite maximizing the imaging depth of the confocal microscope, this method of imaging resulted in low resolution images that are insufficient for visualizing any projections that may occur below the spheroid or for accurately determining the depth of the projections. We note that PBS was used to mount the coverslip and images were captured using an air lens rather than an immersion lens. However, we wanted to make a direct comparison between optical z-stacks captured using these parameters and the method outlined below.

To improve deep tissue visualization, we embedded brain slices containing GFP-expressing tumour spheroids into agar (**Figure 1C**), which allowed us to generate 200 µm sections perpendicular to the brain slice using the vibratome (**Figure 3B**). Each brain slice generates approximately 30 sections. The 200 µm sections were collected in order from top to bottom of the brain slice, flipped 90 degrees and rotated clockwise, and then placed flat onto a microscope slide. The confocal microscope was then used to image invasion into the brain slice in the Z-direction without the requirement for mounting medium or multiple Z-stack images (**Figure 3B**). We suggest implanting tumour spheroids on at least 3 different brain slices collected from the same brain to produce technical replicates. Averaging the area of invasive edge from these technical replicates will produce a single value for that murine brain. Biological replicates should be produced using 3 or more murine brains collected and processed on different experimental days. Overall, this imaging technique greatly improved the visualization of tumour cells invading deep into the brain slice, particularly cells below the spheroid that would otherwise go undetected using traditional imaging techniques.

**Figure 4:**
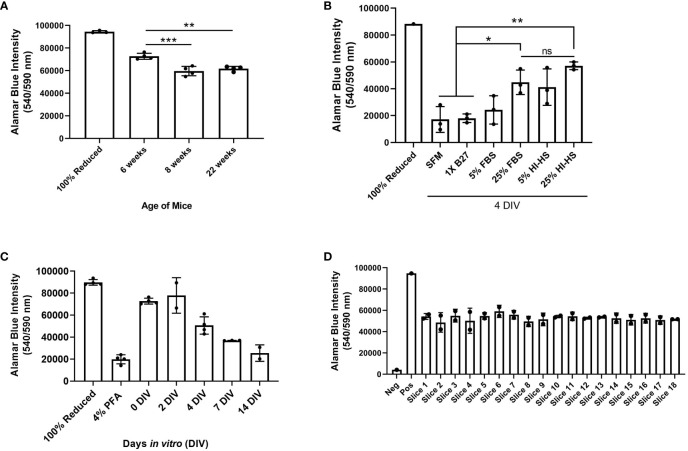
Assessment of brain slice viability in culture. **(A)** Brain slices were collected from 6, 8, or 22 week old mice and cultured for 4 days in vitro (DIV). Cell viability was assessed by incubating brain slices in 10% alamarBlue™ for 24 hours. Autoclaved alamarBlue™ solution (100% reduced) is used as the positive control. Data are mean ± SD (n=4 mice, **p< 0.01, ***p < 0.001, one-way ANOVA with Tukey’s multiple comparison test). **(B)** Brain slices were collected in duplicate from 6-week old male mice and cultured in serum-free medium (SFM), medium containing 1X B27 supplement, DMEM containing 5% or 25% fetal bovine serum (FBS), or DMEM containing 5% or 25% heat-inactivated horse serum (HI-HS) for 4 days in vitro (DIV). Data are mean ± SD (n=3 mice, *p< 0.05, **p < 0.01, one-way ANOVA with Tukey’s multiple comparison test). **(C)** Brain slices collected from 6-week old mice were incubated in DMEM containing 5% FBS for 0, 2, 4, 7, and 14 days *in vitro* (DIV) and brain slice viability was assessed *via* alamarBlue™ intensity. Data represent the mean ± SD (n ≥ 2 mice). **(D)** Brain slice viability of each slice collected from 8-week old mice was determined *via* alamarBlue™ intensity at 0 DIV (n=2 mice). 10% alamarBlue™ solution with no brain slice represents the negative control.

We performed a series of experiments to test the viability of *ex vivo* brain slices under several conditions. Brain slices were harvested from 6-week-old, 8-week-old, and 22-week-old male C57BL/6J mice and then slice viability was analyzed using an alamarBlue™ metabolic assay to determine the role of mouse age on slice viability. We found slices harvested from 6-week-old mice were significantly more viable at 0 days *in vitro* (DIV) compared to the older mice (**Figure 4A**). Notably, 0 DIV refers to brain slices that were incubated at 37°C and 5% CO_2_ for 2-4 hours until all slices were harvested, and then incubated in 10% alamarBlue™ containing medium for another 24 hours. While we limited the mouse age to a minimum of 6-weeks old to ensure the brains are large enough to collect at least 6 slices per brain, using younger mice may further increase viability as well. To compare various brain slice culture media, brain slices harvested from 6-week-old male C57BL/6J mice were cultured in serum-free DMEM-F12 medium (SFM), SFM supplemented with 1X B27 (a serum-free neuronal cell supplement), DMEM-F12 medium containing 5% or 25% FBS, and finally DMEM-F12 medium containing 5% or 25% heat-inactivated horse serum (HI-HS) for 4 days *in vitro* (DIV). This analysis revealed that slices maintained in 25% FBS or 25% HI-HS were significantly more viable than slices in serum-free DMEM-F12 medium or serum-free medium containing 1X B27 (**Figure 4B**). We also observed a lack of brain slice integrity and swelling that occurred when cultured in medium lacking FBS or HI-HS (data not shown). Next, we harvested brain slices from 6-week-old male C57BL/6J mice and cultured the slices in 25% FBS DMEM-F12 medium for up to 14 DIV. Brain slice viability was analyzed over time, which revealed that brain slices maintain greater than 50% viability by 4 DIV, as compared to 0 DIV brain slices (**Figure 4C**), and then viability drops substantially by 7 and 14 DIV. Brain slices fixed in 4% PFA were used as a positive control to determine maximum cell death measurable using alamarBlue™. Finally, each brain slice was collected in sequential order from 8-week-old mice to compare the viability of brain slices harvested from different regions of the brain. This revealed no difference in slice viability when comparing the first slice (Slice 1) to the last slice collected (Slice 18), which represent the slices closest to the occipital lobe and cerebellum, respectively (**Figure 4D**).

**Figure 5:**
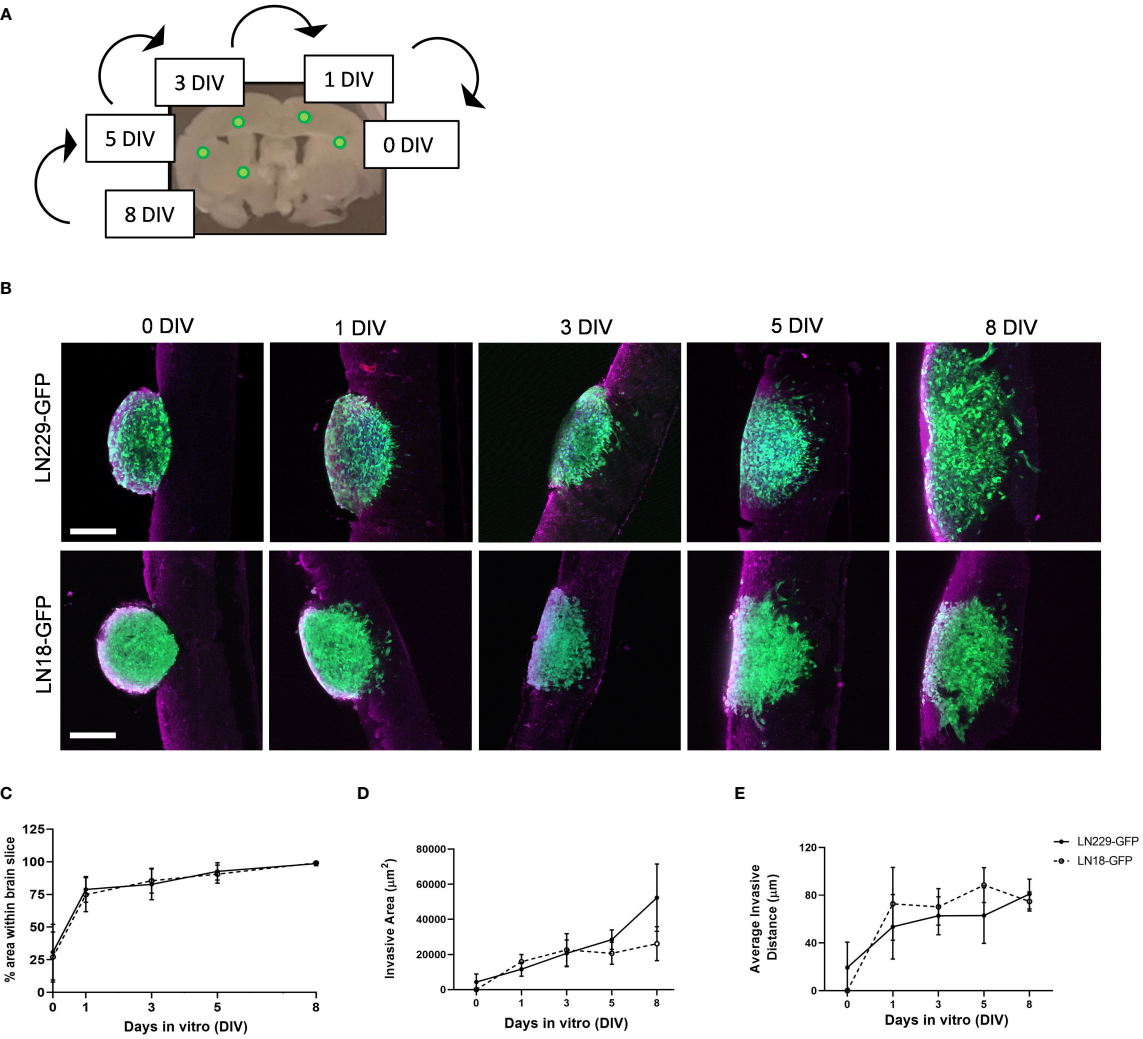
Time course of LN229 and LN18 GFP-tagged GBM spheroids invading into brain tissue *ex vivo*. **(A)** A schematic displaying the approximate location of GFP-tagged spheroids sequentially implanted onto brain slices and cultured *ex vivo* for the indicated amount of time. **(B)** Representative images of LN229-GFP and LN18-GFP tagged GBM spheroids invading into brain slices *ex vivo* for 0, 1, 3, 5, and 8 days *in vitro* (DIV). Brain slices were co-stained for GFAP (astrocytes). Scale bar = 200 µm. **(C–E)** Quantification of the **(C)** % area of spheroid within brain slice, **(D)** the invasive area (µm^2^), and **(E)** the average particle distance from the nearest edge of the ellipse (n= 4 - 8 spheroids per condition implanted on slices collected from ≥4 mice; 3 independent experiments).

To demonstrate the use of our *ex vivo* brain slice invasion assay, we sequentially implanted GFP-expressing LN229 and LN18 spheroids onto murine brain slices between 0 and 8 days prior to imaging. We chose to sequentially implant all spheroids onto the same slice over time to reduce slice-to-slice variability (**Figure 5A**). The time course analysis revealed that approximately 25% of the volume of each LN229-GFP and LN18-GFP spheroid are within the brain slice on day 0 (*i.e.* 2 hours post-implantation), while nearly 100% of the volume of each spheroid was within the brain slice by days 3 and 5, respectively (**Figures 5B, C**). The ImageJ macro (**Figure 2**) was used to quantify the invasive area, which revealed that LN229-GFP spheroids exhibited an increase in the total invasive area, while the invasiveness of LN18-GFP spheroids plateaued by 3 days *in vitro* (DIV) (**Figure 5D**). Furthermore, the average invasive distance, which was determined by measuring the distance of each particle (*i.e* area defined by ImageJ macro as greater than 10 pixels^2^ in size) to the nearest edge of the ellipse, was found to increase up to 1 DIV and then plateaued from 1 DIV to 8 DIV (**Figure 5E**). These data show that our *ex vivo* brain slice invasion assay is sensitive enough to quantify differences in GBM invasion over time between cell lines.

**Figure 6:**
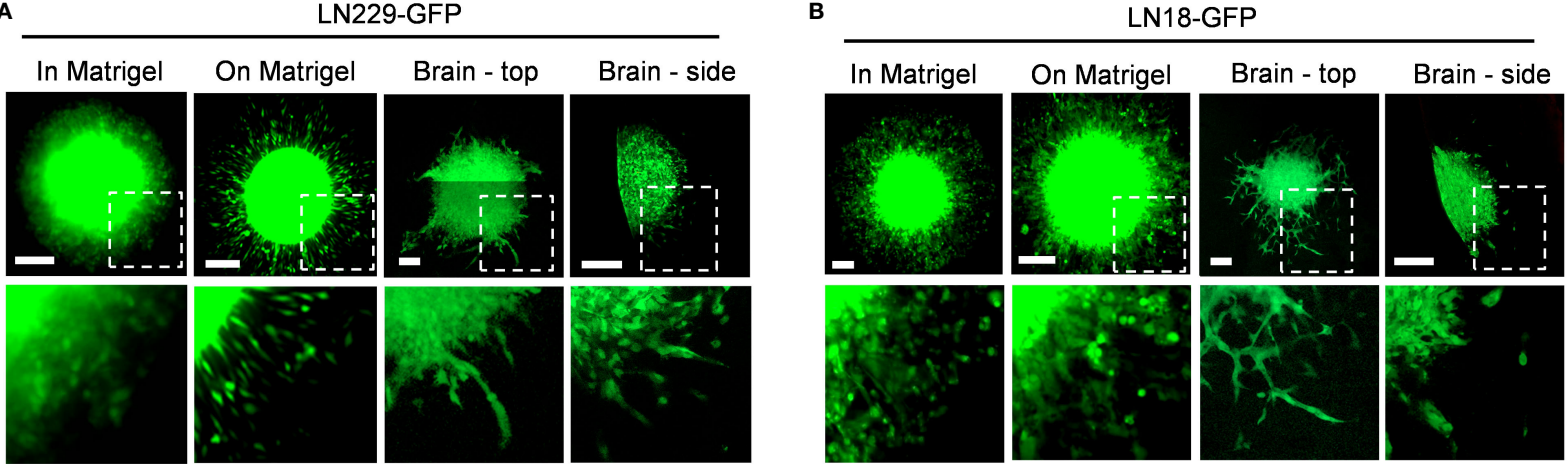
Different invasive phenotypes of GBM cells when embedded into Matrigel versus murine brain tissue. **(A, B)** Representative images of LN229-GFP and LN18-GFP spheroids either embedded within 5.0 mg/mL Matrigel *in vitro* (in Matrigel), seeded on top of a thin layer of Matrigel *in vitro* (on Matrigel), seeded on top of *ex vivo* brain slice and imaged in the X/Y direction (Brain – top), or seeded on top of an *ex vivo* brain slice and imaged in the Z direction (Brain – side) on Day 5. Images of spheroids embedded in or seeded on top of Matrigel were imaged using a Zeiss AxioVert S100 fluorescence microscope with a 10X/0.25 objective. Spheroids seeded on top of brain slices were imaged using the Zeiss LSM 800 confocal microscope with a 20X/0.8 objective. The boxed regions are enlarged below each image. Scale bars = 200 µm.

To directly compare *in vitro* and *ex vivo* models of GBM invasion, we determined the invasiveness of GBM cells when invading into Matrigel versus *ex vivo* brain slices (**Figure 6**). First, LN229-GFP and LN18-GFP spheroids were either embedded in Matrigel or seeded on top of a thin layer of Matrigel. This revealed that both LN229 and LN18 cells invade into the Matrigel or migrate along the top of the Matrigel collectively as sheets of cells (**Figures 6A, B**). Next, LN229-GFP and LN18-GFP spheroids were seeded on top of 300 µm thick organotypic brain slices and cultured *ex vivo.* Imaging the spheroids in the X/Y direction (described in **Figure 3A**) revealed that GBM cells tend to migrate away from the edge of the spheroids collectively as both single cells and spindle-like structures along the top of the brain slice (**Figures 6A, B**). Similarly, imaging in the Z-direction (as described in **Figure 3B**) shows a combination of single cells and spindle-like strands invading into the brain slice (**Figures 6A, B**). These data indicate GBM invasion into Matrigel *in vitro* does not accurately model the invasive behaviour of GBM cells into the brain, suggesting *ex vivo* brain slices are more suitable for studying mechanisms of GBM cell invasion.

**Figure 7:**
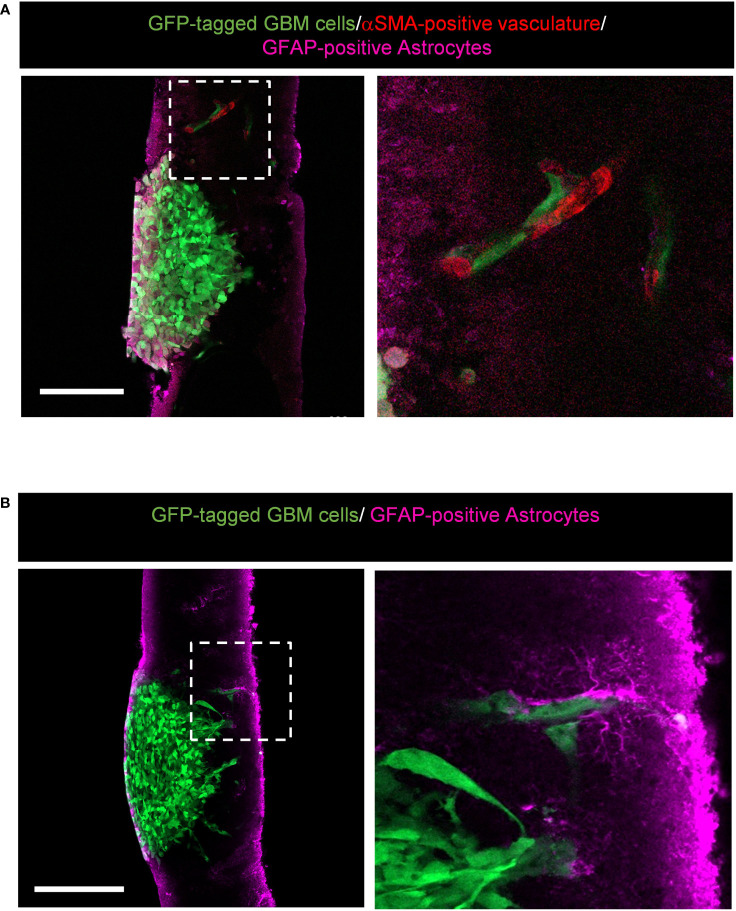
Representative images displaying potential interactions between alpha-smooth muscle actin (αSMA)-positive vasculature and glial fibrillary acidic protein (GFAP)-positive astrocytes within the brain environment. **(A)** High resolution confocal images of GFP-tagged LN229 GBM spheroids invading into murine brain tissue sections stained for GFAP (astrocytes in pink) and αSMA (vasculature in red). The boxed region is enlarged to the right of the image. Scale bar = 200 µm. **(B)** High resolution confocal images of GFP-tagged LN229 GBM spheroids invading into murine brain tissue sections stained for GFAP (astrocytes, pink). The boxed region is enlarged to the right of the image. Scale bar = 200 µm.

Our version of the *ex vivo* brain slice invasion assay can also capture GBM tumour cells interacting with the surrounding brain environment. We found that GFP-tagged GBM cells invading away from the edge of the tumour spheroid can interact with αSMA-positive vasculature (**Figure 7A**) and GFAP-positive astrocytes (**Figure 7B**). These interactions would not be visible by imaging brain slices in the Z-direction using top-down confocal methods, and our system therefore provides the opportunity to more readily study glioma invasion along brain-specific structures.

## 4 Discussion

For decades, GBM cell invasion has typically been modeled *in vitro* using chemotactic assays and synthetic basement membranes. These traditional assays lack environmental influences and may not accurately model the precise mechanisms of GBM cell invasion. Thus, there is a great need to transition from overly simplistic *in vitro* models to utilizing more physiologically relevant model systems to study GBM invasion, which will undoubtedly improve the development of more effective therapeutics for GBM patients. Here, we outline how to perform an *ex vivo* brain slice assay to more quantitatively model brain cancer invasion. Our version of the *ex vivo* brain slice invasion assay greatly improves upon the imaging techniques used to visualize invasion into the brain slice. This technique involves allowing GBM cells to invade into brain slices from spheroids, staining the brain slices with GFAP or other markers, embedding the stained slices in agar, and then re-sectioning the brain slices onto slides to allow visualization of the spheroid-brain slice interface in the Z-direction.

Our method provides an improved workflow for imaging tumour cell invasion depth using a confocal microscope, although we did not compare our methodology to imaging brain slices with a two-photon microscope. Two-photon microscopy is an alternative to confocal microscopy that offers increased resolution when imaging thick tissue sections, and can image tissue at depths that are unachievable using a confocal microscope (20). However, two-photon microscopes are highly specialized and expensive machines that are inaccessible to many labs. Alternatively, optical imaging depth can be improved by chemically clearing fixed tissue using a variety of treatment methods. Tissue clearing methods result in transparent tissue and increased imaging depth since light from the confocal microscope can penetrate into much thicker samples (21–23). Major disadvantages of tissue clearing include the use of harsh organic solvents, which may result in loss of lipids over time and tissue shrinkage, as well as fluorescent quenching (24, 25). Furthermore, tissue clearing processing can take weeks to complete. Our method offers a faster and more accessible way to image brain slices while maintaining staining quality and tissue architecture, as well as allowing quantification of tumour cell invasion.

Organotypic models of invasion offer many advantages over *in vitro* and *in vivo* assays. Seeding spheroids onto brain slices *ex vivo* requires far less technical expertise than performing intracranial implants to establish brain tumours in mice. Furthermore, less mice are required to perform *ex vivo* versus *in vivo* experiments highlighting *ex vivo* models as more cost-effective and ethical options (26). Lastly, organotypic brain slice cultures offer a 3D scaffold that contains tissue-specific ECM and normal cell populations that are often not present within *in vitro* models. Notably, others have shown that organotypic slices collected from fresh pancreatic ductal adenocarcinoma tumours could be cultured *ex vivo* for one week while maintaining immune cell populations of macrophages and T cells (27). While there are many benefits to using organotypic models of invasion, limitations still exist. For instance, while brain slices maintain vascular architecture *ex vivo*, the vessels do not maintain blood flow thus limiting the possibility of immune cell infiltration and tumour cell extravasation. Other limitations of *ex vivo* slice cultures include viability-dependent time constraints as well as the need to purchase specialized equipment (*i.e.*, vibratome). Despite these limitations, *ex vivo* brain slice invasion assays offer many advantages over traditional two-dimensional chemotactic invasion assays and represent an important step forward for GBM research.

Glioblastoma tumours are highly invasive and typically invade within 1-2 cm from the primary tumour margins while rarely metastasizing to distant organs (8, 28). Notably, GBM cells can invade into brain tissue as single cells and/or in collective strands along pre-existing brain structures such as myelinated axons, blood vessels, or white matter tracts (7, 9, 11, 29–31). To determine the best method for modelling these distinct mechanisms of GBM cell invasion, we compared the invasive patterns of GBM cells when using an *in vitro* spheroid invasion assay and an *ex vivo* brain slice invasion assay. Interestingly, we found that LN229 and LN18 GFP-expressing GBM spheroids typically undergo collective invasion with cells invading in a diffuse, sheet-like pattern when either seeded on top of a thin layer of Matrigel or embedded within the Matrigel *in vitro*. Conversely, when the same GBM spheroids were implanted on top of murine brain slices *ex vivo*, we observed GBM cells migrating along the top of the slice and invading into the brain slice as both single cells and in strands of cells. These data therefore suggest *in vitro* models of invasion are lacking critical components necessary to facilitate collective invasion and that modeling GBM invasion using *ex vivo* brain slices better mimics the modes of invasion observed in human GBM tumours.

A major advantage of the organotypic brain slice invasion assay is the ability to implant cell line-derived or patient-derived tumour spheroids anywhere on the brain slice with a high level of precision (*i.e*. spheroids can be implanted only along the corpus collosum, only within white matter, etc.). The invasion assay presented herein models tumour cell invasion within the cerebral cortex, although this model could theoretically be used to evaluate invasion within the occipital lobe or cerebellum as well. However, we do not recommend using this method to model meningeal invasion since there is limited meningeal surface area present in the coronal brain slice sections. Also, we have found spheroids placed along the edge of the brain can detach and/or produce tumour cell migration along the outside of the brain slice and onto the membrane beneath the brain slice. Nevertheless, the *ex vivo* brain slice invasion assay also offers a high-throughput and time-effective option for measuring the effects of genetic manipulation (*i.e.*, siRNA, shRNA, CRISPR/cas9, etc.) or therapeutic intervention (*i.e.*, chemotherapy, immunotherapy, or radiation studies) on GBM cell invasion. For instance, tumour spheroids can either be pre-treated or exposed to various therapeutics once implanted onto the brain slice to analyze the effects of treatment on cancer cell survival, proliferation, and motility. Lastly, this model also provides the ability to study tumour cell-brain cell interactions, such as tumour cells interacting with astrocytes and/or nearby vasculature.

Taken together, our data highlight the importance of transitioning from *in vitro* models to organ-specific *ex vivo* models of invasion to better recapitulate features of the innate tumour microenvironment and surrounding tissue environment. This will result in an improved understanding of the precise mechanisms that drive GBM cell invasion and lead to the advancement of therapeutic options available to brain cancer patients.

## Data availability statement

The raw data supporting the conclusions of this article will be made available by the authors, without undue reservation.

## Ethics statement

The animal study was reviewed and approved by University of British Columbia (UBC) Animal Care Committee in accordance with Canadian Council on Animal Care Guidelines.

## Author contributions

LD, CP, and KB conceptualized the project. LD designed and performed the experiments, analyzed and interpreted the data presented, and wrote the final manuscript. RS conceptualized and wrote the ImageJ macro code for analyzing brain slice invasion. KT assisted with fixing brain slices and MH helped generate tumour spheroids for implantation. CP and KB secured funding and edited the final manuscript. All authors contributed to the article and approved the submitted version.

## References

[1] Thakkar JP Dolecek TA Horbinski C Ostrom QT Lightner DD Barnholtz-Sloan JS . Epidemiologic and molecular prognostic review of glioblastoma. Cancer Epidemiol Biomarkers Prev (2014) 23:1985–96. doi: 10.1158/1055-9965.EPI-14-0275 PMC4185005 25053711

[2] Mason WP Del MR Eisenstat D Forsyth P Fulton D Laperrière N . Canadian Recommendations for the treatment of glioblastoma multiforme. Curr Oncol (2007) 14:110–7. doi: 10.3747/co.2007.119 PMC1899357 17593983

[3] Smith T Yuan Y Walker EV Davis FG . Brain tumour registry of Canada (BTRC): Survival Report 2010-2015. Brain Tumour Registry of Canada (BTRC). A Surveillance Res Collab (2019). Available at: https://braintumourregistry.ca/survival-report.

[4] Yuan Y Shi Q Li M Nagamuthu C Andres E Davis FG . Canadian Brain cancer survival rates by tumour type and region: 1992-2008. Can J Public Health (2016) 107:37–42. doi: 10.17269/CJPH.107.5209 PMC6972338 27348108

[5] Cha J Kim P . Biomimetic strategies for the glioblastoma microenvironment. Front Mat (2017) 4:45/BIBTEX. doi: 10.3389/FMATS.2017.00045/BIBTEX

[6] Lau LW Cua R Keough MB Haylock-Jacobs S Yong VW . Pathophysiology of the brain extracellular matrix: a new target for remyelination. Nat Rev Neurosci (2013) 14:722–9. doi: 10.1038/nrn3550 23985834

[7] Scherer HJ . Structural development in gliomas. Am J Cancer (1938) 34:333–51. doi: 10.1158/AJC.1938.333

[8] Esmaeili M Stensjøen AL Berntsen EM Solheim O Reinertsen I . The direction of tumour growth in glioblastoma patients. Sci Rep (2018) 8:1199. doi: 10.1038/s41598-018-19420-z 29352231 PMC5775193

[9] Liu CJ Shamsan GA Akkin T Odde DJ . Glioma cell migration dynamics in brain tissue assessed by multimodal optical imaging. Biophys J (2019) 117:1179–88. doi: 10.1016/j.bpj.2019.08.010 PMC6818150 31474305

[10] Cuddapah VA Robel S Watkins S Sontheimer H . A neurocentric perspective on glioma invasion. Nat Rev Neurosci (2014) 15:455–65. doi: 10.1038/NRN3765 PMC5304245 24946761

[11] Mair DB Ames HM Li R . Mechanisms of invasion and motility of high-grade gliomas in the brain. Mol Biol Cell (2018) 29:2509–15. doi: 10.1091/MBC.E18-02-0123 PMC6254577 30325290

[12] Ohnishi T Matsumura H Izumoto S Hiraga S Hayakawa T . A novel model of glioma cell invasion using organotypic brain slice culture. Cancer Res (1998) 58:2935–40. 9679949

[13] Gritsenko P Leenders W Friedl P . Recapitulating *in vivo*-like plasticity of glioma cell invasion along blood vessels and in astrocyte-rich stroma. Histochem Cell Biol (2017) 148:395–406. doi: 10.1007/s00418-017-1604-2 28825130 PMC5602046

[14] Neve A Kumar KS Tripolitsioti D Grotzer MA Baumgartner M . Investigation of brain tissue infiltration by medulloblastoma cells in an ex vivo model. Sci Rep (2017) 7:1–12. doi: 10.1038/s41598-017-05573-w 28706234 PMC5509741

[15] Jung S Kim HW Lee JH Kang SS Rhu HH Jeong YIL . Brain tumor invasion model system using organotypic brain-slice culture as an alternative to *in vivo* model. J Cancer Res Clin Oncol (2002) 128:469–76. doi: 10.1007/S00432-002-0366-X PMC12164502 12242510

[16] Eisemann T Costa B Strelau J Mittelbronn M Angel P Peterziel H . An advanced glioma cell invasion assay based on organotypic brain slice cultures. BMC Cancer (2018) 18:103. doi: 10.1186/s12885-018-4007-4 29378533 PMC5789681

[17] Grabiec U Hohmann T Hammer N Dehghani F . Organotypic hippocampal slice cultures as a model to study neuroprotection and invasiveness of tumor cells. J Visualized Exp (2017) 2017:e55359. doi: 10.3791/55359 PMC5614375 28872113

[18] Pencheva N de Gooijer MC Vis DJ Wessels LFA Würdinger T van Tellingen O . Identification of a druggable pathway controlling glioblastoma invasiveness. Cell Rep (2017) 20:48–60. doi: 10.1016/j.celrep.2017.06.036 28683323

[19] Chuang HN Lohaus R Hanisch UK Binder C Dehghani F Pukrop T . Coculture system with an organotypic brain slice and 3D spheroid of carcinoma cells. J Vis Exp (2013) 80:50881. doi: 10.3791/50881 PMC3939073 24145580

[20] Benninger RKP Piston DW . Two-photon excitation microscopy for the study of living cells and tissues. Curr Protoc Cell Biol (2013) 59:4.11.1–4.11.24. doi: 10.1002/0471143030.CB0411S59 PMC4004770 23728746

[21] Ke MT Fujimoto S Imai T . SeeDB: a simple and morphology-preserving optical clearing agent for neuronal circuit reconstruction. Nat Neurosci (2013) 16:1154–61. doi: 10.1038/nn.3447 23792946

[22] Hama H Hioki H Namiki K Hoshida T Kurokawa H Ishidate F . ScaleS: an optical clearing palette for biological imaging. Nat Neurosci (2015) 18:1518–29. doi: 10.1038/nn.4107 26368944

[23] Hama H Kurokawa H Kawano H Ando R Shimogori T Noda H . Scale: a chemical approach for fluorescence imaging and reconstruction of transparent mouse brain. Nat Neurosci (2011) 14:1481–8. doi: 10.1038/nn.2928 21878933

[24] Gómez-Gaviro MV Sanderson D Ripoll J Desco M . Biomedical applications of tissue clearing and three-dimensional imaging in health and disease. IScience (2020) 23:101432. doi: 10.1016/J.ISCI.2020.101432 32805648 PMC7452225

[25] Ariel P . A beginner’s guide to tissue clearing. Int J Biochem Cell Biol (2017) 84:35. doi: 10.1016/J.BIOCEL.2016.12.009 28082099 PMC5336404

[26] Vollmann-Zwerenz A Leidgens V Feliciello G Klein CA Hau P . Tumor cell invasion in glioblastoma. Int J Mol Sci (2020) 21:1932. doi: 10.3390/IJMS21061932 32178267 PMC7139341

[27] Jiang X Seo YD Sullivan KM Pillarisetty VG . Establishment of slice cultures as a tool to study the cancer immune microenvironment. Methods Mol Biol (2019) 1884:283–95. doi: 10.1007/978-1-4939-8885-3_20 30465211

[28] Choucair AK Levin VA Gutin PH Davis RL Silver P Edwards MS . Development of multiple lesions during radiation therapy and chemotherapy in patients with gliomas. J Neurosurg (1986) 65:654–8. doi: 10.3171/JNS.1986.65.5.0654 3021931

[29] Alieva M Leidgens V Riemenschneider MJ Klein CA Hau P van Rheenen J . Intravital imaging of glioma border morphology reveals distinctive cellular dynamics and contribution to tumor cell invasion. Sci Rep (2019) 9:1–11. doi: 10.1038/s41598-019-38625-4 30765850 PMC6375955

[30] Claes A Idema AJ Wesseling P . Diffuse glioma growth: A guerilla war. Acta Neuropathol (2007) 114:443–58. doi: 10.1007/S00401-007-0293-7/FIGURES/3 PMC2039798 17805551

[31] Haeger A Krause M Wolf K Friedl P . Cell jamming: Collective invasion of mesenchymal tumor cells imposed by tissue confinement. Biochim Biophys Acta (BBA) - Gen Subj (2014) 1840:2386–95. doi: 10.1016/J.BBAGEN.2014.03.020 24721714

