# Supplementary material for: Development and validation of an advanced ex vivo brain slice invasion assay to model glioblastoma cell invasion into the complex brain microenvironment
Source: Front Oncol. 2023 Jan 26;13:976945. doi: 10.3389/fonc.2023.976945 (PMC9923402; doi:10.3389/fonc.2023.976945)
Supplement: Supplementary file 1 [file DataSheet_1.pdf]

Supplemental Methods

Generation of GFP-expressing LN229 and LN18 cells

HEK293TD cells were co-transfected with pLenti CMV GFP Blast, pMD2.G, and psPAX2 plasmid constructs (pLenti CMV GFP Blast (659-1) was a gift from Eric Campeau & Paul Kaufman, pMD2.G and psPAX2 were gifts from Didier Trono; Addgene plasmid # 17445, #12259, and #12260, respectively) using Lipofectamine 2000 (11668019, ThermoFisher). After overnight incubation, the supernatant carrying the virus was filtered (0.45µm) to remove cell debris and 8 µg/mL of Polybrene (H9268, Hexadimethrine bromide, Millipore Sigma) was subsequently added. The mixture was then added to the LN229 and LN18 cells. The plates with LN229 and LN18 cells and the media containing virus were spun down at 1200 x g for 60 min at 32°C to aid with transduction efficiency. The cells were incubated overnight and then selected with 8µg/mL of Blasticidin S (R21001, ThermoFisher) for 3 days.
